# Alcohol brief interventions in Scottish antenatal care: a qualitative study of midwives’ attitudes and practices

**DOI:** 10.1186/1471-2393-14-170

**Published:** 2014-05-21

**Authors:** Lawrence Doi, Helen Cheyne, Ruth Jepson

**Affiliations:** 1 Scottish Collaboration for Public Health Research and Policy, Centre for Population Health Sciences, University of Edinburgh, Scotland, UK; 2 Nursing, Midwifery and Allied Health Professionals Research Unit, University of Stirling, Scotland, UK

**Keywords:** Midwives, Pregnant women, Screening and alcohol brief interventions, Fetal harm, Qualitative research

## Abstract

**Background:**

Infants exposed to alcohol in the womb are at increased risk of experiencing health problems. However, mixed messages about the consequences of prenatal alcohol consumption have resulted in inconsistent attitudes and practices amongst some healthcare practitioners. Screening and alcohol brief interventions (ABIs) can reduce risky drinking in various clinical settings. Recently, a program of screening and ABIs have been implemented in antenatal care settings in Scotland. However, current evidence suggests that midwives’ involvement in alcohol brief interventions activities is patchy. This study explored midwives’ attitudes and practices regarding alcohol screening and ABIs in order to understand why they are relatively underutilized in antenatal care settings compared to other clinical settings.

**Methods:**

This was a qualitative study, involving semi-structured interviews with 15 midwives and a focus group with a further six midwifery team leaders (21 participants in total) in Scotland. Interview transcripts were analysed using thematic analysis.

**Results:**

Midwives were positive about their involvement in the screening and ABI program. However, they were not completely convinced about the purpose and value of the screening and ABIs in antenatal care. In the midst of competing priorities, the program was seen as having a low priority in their workload. Midwives felt that the rapport between them and pregnant women was not sufficiently established at the first antenatal appointment to allow them to discuss alcohol issues appropriately. They reported that many women had already given up drinking or were drinking minimal amounts prior to the first antenatal appointment.

**Conclusions:**

Midwives recognised the important role they could play in alcohol intervention activities in antenatal care. As the majority of women stop consuming alcohol in pregnancy, many will not need an ABI. Those who have not stopped are likely to need an ABI, but midwives were concerned that it was this group that they were most likely to alienate by discussing such concerns. Further consideration should be given to pre-pregnancy preventative measures as they are more likely to reduce alcohol-exposed pregnancies.

## Background

Infants exposed to alcohol in the womb are at increased risk of experiencing health problems, including neuro-developmental conditions and fetal alcohol syndrome [1-3]. Children born with developmental disabilities have long-term health and social care requirements, which may place a considerable resource burden on families and the health services. Therefore, interventions that aim to prevent alcohol use in pregnancy have the potential to improve children’s developmental outcomes. Screening and alcohol brief interventions (ABIs) can reduce alcohol consumption or encourage abstinence in individuals drinking at hazardous and harmful levels [4,5]. Alcohol brief interventions are time-limited interventions that can range from a single 10–15 minutes session to several sessions of assessment, personalised feedback about drinking behaviour, goal setting, behaviour modification strategy and minimal follow-up reinforcement visits or on-going support [6,7]. Current evidence supports the use of screening and ABIs to reduce hazardous and harmful drinking in a range of healthcare settings, including primary care and general hospital settings [8,9]. However, only a limited number of studies have been conducted with antenatal populations therefore, evidence of their effectiveness is still evolving in this setting [10,11].

Typically, the delivery of ABIs is preceded by the use of alcohol screening tools to detect hazardous or harmful drinking. These tools are usually setting and population-specific. Validated alcohol screening tools such as T-ACE (Tolerance, Annoyed, Cut- down, Eye-opener) and TWEAK (Tolerance, Worried, Eye-opener, Amnesia, (K) Cut-down) are usually recommended for screening pregnant women because they have high sensitivity and specificity for this population group [12-14]. In Scotland, national guidelines suggest that if a woman is drinking alcohol in pregnancy, an ABI should be delivered without further screening unless there is reason to suspect possible dependence, in which case a referral should be made to a more specialist service or practitioner [15]. The guidelines are in line with the current National Health Services (NHS) Health Scotland advice to pregnant women, which states that it is best to avoid alcohol completely during pregnancy, as any alcohol taken while pregnant will reach the baby and may cause harm [16]. Midwives are encouraged to inquire about alcohol consumption at the first antenatal appointment. When women respond positively, the number, nature and size of drinks consumed are explored in order to estimate alcohol units [15].

Scotland’s alcohol-related health burden is among the highest in Europe [17]. In order to reduce this, the Scottish Government established a health improvement target for NHS Health Boards, requiring them to screen and deliver a total of 149,449 ABIs from April 2008 to March 2011 in the priority settings of primary care, accident and emergency departments and antenatal care, with the aim of incorporating them into routine clinical practice [18,19]. In order to support workforce development, practitioners involved in screening and delivering the ABIs were trained [20]. An ABI delivery support team was also established to provide expert advice to stakeholders on the actions required to achieve the target. They also provided guidance and strategic leadership to NHS Health Boards in terms of the development of the delivery infrastructure and implementation of ABIs [21].

In antenatal care settings, midwives have been given the role of screening and delivering ABIs to pregnant women because within the UK they provide the majority of antenatal care to women. However, current knowledge regarding midwives’ involvement in alcohol intervention activities is limited [22]. There are also uncertainties regarding the effects of prenatal alcohol consumption on the fetus, particularly at low-moderate levels of drinking [23]. This may have led midwives to have a range of opinions about the issue of alcohol use in pregnancy. In order to understand and improve the quality of screening and ABIs in antenatal care settings, we explored how midwives’ skills, knowledge and attitudes to alcohol consumption in pregnancy influence their practice.

## Methods

### Sample and procedure

NHS care in Scotland is provided through 14 regional Health Boards, which plan and deliver health services within their geographical areas, with overall policy directed by the Scottish Government Health Directorates [24]. Participants for this study were recruited from one Scottish Health Board, NHS Lothian, which has two consultant units and about 130 community midwives overall. Our sample of community midwives was drawn from the 34 midwives based at one of the consultant units. Within NHS Scotland, community based midwives provide the majority of care to women during the antenatal and post-natal periods, involving the women’s General Practitioner or Obstetrician as appropriate based on the woman’s needs and local care pathways [25]. As such, they are well placed to screen and deliver ABIs to pregnant women. At the time of data collection in mid to late 2010, the midwives who participated in this study informed us that nearly all midwives in NHS Lothian, particularly community midwives, were trained to screen and deliver ABIs. The training involved half a day face-to-face screening and ABI skill training. All community midwives involved in alcohol screening and delivery of ABIs were considered for inclusion. This study purposively sampled midwives with a range of roles - community midwives (middle grade clinical midwives) and their team leaders, as well as consultant midwives (senior clinical midwives with a remit for professional leadership, evaluation and education). Details of the study in the form of information packs were delivered to three midwifery team leaders to be given to all midwives within their areas. The information packs contained an invitation letter, information sheet (including details of incentives), an expression of interest form and self-addressed stamped envelope. All midwives were given the option of individual interviews or focus group to encourage participation. Informed consent was obtained prior to interview or focus group. Participants were compensated for their time and effort with either lunch or £20 in high street store vouchers.

Semi-structured interviews were used and the topic guide (available on request) covered a range of areas including, attitudes to alcohol use in pregnancy and opinions about the appropriate timing for screening and delivering ABIs. Midwives were encouraged to describe their practice in relation to screening and delivering of ABIs and to identify barriers and facilitators. Their views about training, support and the implementation process were also sought. All interviews and the focus group were conducted and digitally recorded by the first author, who also transcribed the data. The second and third authors reviewed the transcripts. Anonymous unique codes were used to represent midwives who participated in the study.

### Data analysis

The interview transcripts were analysed using a thematic analysis which employed a hybrid approach of inductive and deductive coding and theme development [26]. This combined the data-driven inductive approach [27] and deductive a priori code template [28]. Data analysis proceeded in six main stages as follows: development of a code manual; test reliability of codes; identification of initial themes emerging from the data; utilization of the code manual to apply codes to entire transcripts whilst noting emerging codes; connection of codes into themes and corroboration of themes through interpretation of analysis [26].

For a code to be credible, it must capture the qualitative richness of the phenomenon [27]. Using NVivo [29] coding was undertaken by selecting the appropriate segments of text and coding them appropriately. Several codes were generated. New codes were devised as new ideas emerged from the data. When new codes were identified, previous transcripts were re-read to determine if the new codes were applicable to the texts. Thus, the code manual was continually revised. A final code manual was then produced. The reliability of coding is enhanced if two or more researchers independently code a qualitative transcript [30]. For that reason, the first author coded all transcripts and the second and third authors coded half of the transcripts. Generally, inter-coder agreements were high, with over 80% agreement on coded extracts. Minor disagreements were resolved through discussion between the authors. Through the connection of similar codes, themes emerged. Agreements and disagreements in opinions between segments of the data were informative at this stage. It was also important to ensure that themes were representative of the original data [26]. Consequently, transcripts were re-read ensuring that themes had appropriately captured their phenomena. With the emergence of patterns of meaning, similarities and differences within units of the data were explored before the analysis progressed to the interpretative phase.

### Ethics approval

The West of Scotland Research Ethics Committee 2 approved this study.

## Results

Twenty-five midwives initially expressed an interest to participate in the study however, four subsequently declined, citing time constraints as the main reason. In all, twenty-one midwives participated in the study. Fifteen midwives agreed to participate in face-to-face interviews and six midwifery team leaders participated in a focus group. The semi-structured face-to-face interviews lasted between 35 and 71 minutes while the focus group lasted an hour. As no new themes were emerging with this number of participants, we felt data saturation had been reached.

### Participants’ characteristics

Midwives ages ranged between 24 and 56 years. All were clinical midwives, eight were midwifery team leaders and two were consultant midwives. Their years of midwifery practice ranged between 3 and 33.

### Themes

Seven major themes were identified. Individual interview and focus group data are presented together.

### Attitudes to alcohol consumption

Prompted by previous research findings that there were uncertainties regarding the effects of low-moderate levels of drinking on the fetus, we explored how this message had influenced midwives’ attitudes to prenatal alcohol consumption. We anticipated that midwives’ attitudes may either directly or inadvertently influence their decisions about the advice they offered to women, or the manner in which they delivered ABIs. It was apparent that uncertainties regarding the threshold at which alcohol could harm the baby prompted many of the midwives to take a cautious stance in relation to advising abstinence from alcohol during pregnancy (see Table 1). During the interview, midwives sometimes spoke about their own alcohol use to emphasize a point or convey an opinion and this was used to understand their attitudes towards the issue. Most of the midwives who indicated that they did not drink alcohol had strong views against drinking in pregnancy while a few of the midwives who indicated that they did drink alcohol appeared sceptical regarding the current push for abstinence in pregnancy.

**Table 1:** Midwives’ attitude to drinking

**Sub-theme**	**Illustrative quote**
Influence of uncertainties	I think I have always taken the view that it will be better not to drink at all than be unsure about what amount is safe (M11).
Scepticism	Certainly, as a midwife I will probably join in and say, how come women in other countries drink and they don’t suffer from alcohol and here in Britain they are giving mixed messages to women saying don’t drink alcohol at all because it might have an effect on the baby (M9).

### Midwives’ perception of risk

We hypothesized that midwives’ knowledge and understanding of risk could have an influence on the level of priority they accorded to the issue of drinking in pregnancy in the midst of other competing work pressures. Generally, midwives showed a good understanding of the effects of alcohol on the fetus. All the participants knew that heavy sustained drinking is associated with fetal alcohol syndrome. However, there were divided opinions regarding the effects of low-moderate level of drinking on the fetus. Drinking at all stages in pregnancy poses varying risks for the mother and the unborn child [31,32]. We further explored midwives views on the impact of timing of alcohol consumption during pregnancy on subsequent developmental outcomes. It was clear that most considered drinking in the first trimester to be more risky, which is congruent with the evidence [33,34]. However, a few thought that drinking in the first trimesters possibly posed the least risk to the fetus (see Table 2). They reasoned that quite a lot of women had consumed alcohol during the early stages of pregnancy while they were unaware that they were pregnant, yet it appeared that their children were rarely harmed.

**Table 2:** Risk perception

**Sub-theme**	**Illustrative quote**
Effects of low-moderate drinking	Excessive alcohol is always going to be damaging to the woman and the baby. But whether or not if you have a glass of wine with your dinner two or three nights a week if that is going to affect the baby or not, I will probably debate. Probably, say that it wouldn’t have any effect on that baby (M2).
Drinking in first trimester	I don’t know if we really know what the effects of alcohol are on the developing fetus. I am not too sure about that. I think it is more to do with continuing drinking through pregnancy. I mean if you drank alcohol without knowing that you have conceived or in early times of conception, it doesn’t seem to affect (the baby). I would have said that it doesn’t affect (the baby) because so many people have done it, you know (M14).

### Timing of the alcohol brief interventions

The midwives mentioned that most of the pregnant women who drank alcohol did so during the period when pregnancy was unconfirmed. Once pregnancy was confirmed, they discontinued taking alcohol. They all agreed that pre-conception alcohol prevention strategies would be more beneficial to the fetus than alcohol interventions delivered in antenatal care. However, midwives recognised that without a specific target audience such interventions might struggle to have an impact. Some participants expressed the opinion that pregnant women who planned their pregnancies were more likely to benefit from pre-pregnancy interventions (see Table 3). Planning of pregnancy could mean that women would have been more likely to have explored ways to maximise their chances of having a healthy pregnancy. For example, this could involve contacts with family planning clinics regarding advice on discontinuing contraceptive use or contacts with fertility clinics. Midwives believed that some of these pre-pregnancy opportunities would have been more appropriate ‘teachable moments’ because of their emphasis on health issues, particularly those of the unborn child.

**Table 3:** Pre-pregnancy alcohol intervention opportunities

**Sub-theme**	**Illustrative quote**
Few women drink in pregnancy	I think by the time most of them have come to us most of them say I don’t drink now. So they already know that drinking in pregnancy is not recommended (M9).
Pre-pregnancy opportunities	A lot of my clients have gone through the fertility treatment. So you have got a high level of people who are looking for a healthy baby and will have researched it all and would have been given all the information as they go through the IVF program. They have been told there that alcohol use is out of the window (M1).

### Identification and delivery of ABIs

Almost all the midwives indicated that tackling alcohol use in pregnancy was part of their role. Nevertheless, some mentioned that the screening and ABI program had been added to their already considerable workload. One of the rationales for the program was to train midwives to intervene with women who drank at risky levels, but for whom drinking had not reached the dependence level that might require alcohol specialist’s attention. However, a few midwives misconstrued this rationale (see Table 4). There was also a sense that midwives felt that their role was to advise women to be abstinent. This is not entirely in line with the motivational interviewing approach of delivering ABIs, which does not imply a pre-determined decision about what is best for the patient.

**Table 4:** Screening and ABI delivery

**Sub-theme**	**Illustrative quote**
Role legitimacy	The role is constantly growing whereas the midwife’s capacity probably isn’t growing in time with that. But I think midwives see health promotion and public health as part of their role (M15).
Misconception of screening and ABI initiative	To me it (ABI) means their alcohol consumption is unsafe and this is the recommendations, that is to me what a brief intervention is and if it is unsafe then we are going to refer on that’s part of it (M18, FG).
	I think it is good to raise awareness about alcohol and that all we are doing, we are not doing any particular ABI in trying to cure them or anything that is not my job. I am here to educate them to be responsible parents and realise that it is not a good thing to do in pregnancy and just to raise awareness of alcohol consumption (M1).
Few ABIs delivered	Fortunately, I have never had anybody since we started it that needed the help (M1).
Missed opportunity	I haven’t come across any problems so far but as I say there is only one girl who has just booked with me and I don’t know but she has admitted to having about eight units every two weeks (M3).
Importance of alcohol screening	People don’t think they’ve got a problem but once you start adding the units up there’s a fair bit there but then they don’t hide it because they didn’t think it was a problem, you get more out of them than you would before when you just wrote down social drinking, no problem (M21, FG).
Additional resources	Because of the confusion with units (of alcohol) we have cup measures and we have slide rules that we got from the alcohol brief interventions team so that we can show women that vodka, “wicked” or whatever is one and half units and not just one unit. Sometimes that is just enough for them to think, oh my goodness I didn’t think it is so many units! (M7).

At the time of data collection most of the midwives had completed their screening and ABIs training at least six months previously, but it was clear that most of them had not had enough opportunities to practice their skills because they perceived that few women admitted to alcohol consumption in pregnancy. However, it appeared that in some instances the opportunity to deliver ABIs at the antenatal appointment was missed.

 The midwives felt that the assessment to tease out the nature and quantity of alcohol consumed by women was beneficial. Previously, no such detailed assessment was conducted and some midwives believed that the program had improved the quality of identification. Further, midwives felt that the additional support and resources provided by the implementing organisation (NHS Health Scotland) had had positive impact. In addition to the ABI skill training, midwives were also provided with an ABI antenatal professional pack. The pack included information about the evidence base of ABIs, screening tools, prompt cards and a drink calculator (for estimating alcohol units).

### Midwife-pregnant woman relationship

Midwives believed that they were well positioned strategically to deliver health information or ABIs, and that the type of services they provide meant women were more likely to respond to them in terms of accepting health behaviour interventions. However, they believed that they had to be careful in order not to alienate women when discussing sensitive alcohol issues.

Most midwives felt that screening and delivery of ABIs at the first antenatal appointment may not be appropriate because of the potential impact it could have on midwife-pregnant woman relationships. They expressed the feeling that there could be issues with trust at the first appointment and that it would be difficult for women to divulge sensitive issues at that point. One midwife likened the issue of trying to identify pregnant woman who have been drinking at risky levels at the first antenatal visit, to that of identifying a domestic violence victim (see Table 5). In spite of this, midwives felt that in the context of antenatal care, the first appointment was the best opportunity to deliver ABIs.

**Table 5:** Midwife-pregnant woman relationship

**Sub-theme**	**Illustrative quote**
Midwives’ good profile	Midwives have good profile, we look after women we are supposed to have lots of knowledge, we are going to help them through the birth of their baby and give them advice in their first few weeks afterwards. We have got a profile that women hopefully take note of (M9).
Careful when discussing sensitive issues	I mean discussing alcohol with somebody sitting in front of you and you don’t want them to feel that they can’t come and see you again (M14).
Lack of rapport at booking appointment	The other thing that makes it difficult is that at booking you have only just met the person. So, you are already asking a lot of personal questions. You probably haven’t ever met her before and then you are required to take action whether it will be for alcohol or gender based violence. It is very difficult but I don’t know when the good time will be, you know. Because by the time you have met her for three or four times, she is already well on in her pregnancy. And that is the longest appointment that you have so that is the most time you have with somebody (M15).
Women unlikely to divulge sensitive information	I am just going back to the issue of domestic violence, if I was to ask a woman, are you violated against? Are you free to go home? Have you ever suffered violence at home? Do you think she is going to tell me when she does not even know me at booking? (M9).

### Benefits of screening and ABIs

Midwives reported that the program had several positive aspects, for not only women and their unborn child but also for the value it had added to their practice. Midwives’ overall view was that screening and ABIs had generated increased awareness about alcohol use in pregnancy. Some viewed their place in antenatal care as appropriate because pregnancy is a time when women are more motivated to change negative health behaviours. However, others indicated that offering ABIs in antenatal care was a bit late. A few midwives suggested that ABIs may not necessarily be of benefit to the current pregnancy but might benefit subsequent pregnancies and future drinking behaviour. One participant suggested that providing ABIs, particularly to women who consumed excessive amounts of alcohol before pregnancy might be helpful in terms of equipping them with knowledge that could facilitate healthy drinking behaviour after they had had their baby (see Table 6).

**Table 6:** Perceived benefits of screening and ABIs

**Sub-theme**	**Illustrative quote**
Pregnancy may facilitate drinking behaviour change	There are not many other opportunities that women are told you shouldn’t be drinking. I think pregnancy is one of the times that women are more than happy to stop drinking, most women are more than happy to stop so it’s probably a good time to do brief intervention (M5).
Positive drinking behaviour change beyond pregnancy	At the moment, we are trying to discourage them from drinking in pregnancy. You are asking about their drinking habits beforehand which hopefully when they’ve not had alcohol for nine months, it’s easier to go back to a safe limit of alcohol than going back to your old habits because you’ve abstained from alcohol for nine months (M13).
Benefits for mother and baby	If she can stop alcohol and smoking and drugs and have a good diet and do all the positive stuff. And when the baby is born, hopefully he will be born into a smoke free home with parents who don’t drink excessively. It is going to give that child a much better chance. Financially, the woman can’t afford because quite often we are talking about a single mother, they don’t have a partner (M2).
Improved confidence to discuss alcohol issues	I think having done the course though, it makes you more confident to be able to ask them that. It is not just a case of oh well have you had a drink, why? You know (M3).

They also highlighted the potential benefits of screening and ABIs to the child, as well as financial benefits to the family. All midwives recognised the added benefits of the program to their practice in relation to their confidence in discussing alcohol-related issues.

### Barriers to implementation of screening and ABIs

Midwives were asked to identify the main challenges that the implementation of the program had had on their practice or some of the difficulties they had experienced since its inception. It was clear that workload pressure and time constraints were the most significant barriers. Midwives indicated that in the midst of competing workload priorities, alcohol interventions remained a low priority. The difficulty of converting different types of alcoholic drinks into standard units was also seen as a problem and in the focus group all participants agreed with that view (see Table 7).

**Table 7:** Challenges of screening and ABI delivery

**Sub-theme**	**Illustrative quote**
Time constraints	Time is almost always a big issue because if ABI is required, it is not just a simple case of she no longer drinks alcohol and we are happy with the plan so no intervention is required. If intervention is required that could eat into your time or the rest of the care for that booking appointment (M7).
Low priority	We’ve got to do domestic violence, alcohol use, smoking, you know and all the stuff. If somebody says I smoke then we have to give them all the literature, the DVD, arrange for referrals. So you can imagine, alcohol is only one of the aspects and sadly it is not the most important one because there is not a lot of evidence there that we have a lot of children who have fetal alcohol syndrome (M9).
Difficulty in unit conversion	Asking people in terms of units per week is quite difficult because first of all you’ve got to work out what the units are and whether it’s a big glass of wine, small glass of wine, strong wine, weak wine, it’s a real nightmare and then just work it out per week rather (M19, FG).
Overload of information at booking appointments	I guess the other thing is the amount of information that women can take on board. You know if you are thinking that you have got another twenty areas of information to give women you know, you wonder well can they take all that in (M2).
Social expectations	People know that it is not good to drink in pregnancy and therefore they don’t always tell you the truth because they know that maybe you disapprove or it will make them feel guilty if they knew that they are honest and told you (M3).

Some midwives were worried that the amount of information provided at the first antenatal appointment may adversely influence pregnant women’s capacity to assimilate all relevant information, in particular when a behaviour change intervention is involved. The social intricacies surrounding the issue of drinking in pregnancy were also stated as a barrier. A few midwives suggested that because drinking in pregnancy is generally considered undesirable in the Scottish culture, this probably discourages women from disclosing their true consumption levels.

## Discussion

This qualitative study focused on midwives’ attitudes towards alcohol consumption during pregnancy and their practices regarding screening and alcohol brief interventions. Drinking alcohol during pregnancy is an emotive topic and discussing it in the antenatal context may present a challenge for midwives. This could be particularly difficult once midwives become aware that a pregnant woman had used alcohol excessively. In a recent primary care based study in Norway, General Practitioners revealed that they were uncomfortable discussing alcohol use with patients because of its emotive nature [35]. The capacity to deal with patients’ reaction to the subject of alcohol consumption has also been noted as discouraging health professionals from initiating alcohol intervention activities [36]. In this study, a few midwives missed opportunities to deliver ABIs and it was possible that they felt that if a pregnant woman had already used excessive alcohol, the harm could not be undone and that discussing it might cause anxiety and distress to her. The findings also suggested that midwives were keen to avoid putting any strain on the pregnant woman-midwife relationship in discussing these issues.

Midwifery care has traditionally had a public health role, recent Scottish Government policy has increased the public health focus of maternity care with the aim of encouraging midwives to assume a greater role in order to improve health and social wellbeing for all women and reduce health inequalities [37,38]. This means that the community midwives’ role has now become ever more demanding as they negotiate complex care priorities. For example, they now have to deal with an increase in birth rate, clinical assessment, increased involvement with assessment of child protection issues, women who have complex health and social care needs, and women who are misusing alcohol and drugs, as well as the increase in numbers of migrant women who require the use of interpreter for antenatal appointments [37]. Delivering these services often requires increased focus on supporting the women and their families [37]. However, some studies have suggested that in spite of midwives being encouraged to provide supportive relationships to clients, they may not always feel adequately prepared for the supportive nature of their role [39,40]. With alcohol intervention activities, lack of training and support for health professionals have largely been identified as significant barriers [36,41,42]. In the current study, midwives were appreciative of the training and support strategies put in place for them. They generally felt confident about their skills to screen and deliver ABIs, although some were quite hesitant about the program’s usefulness in terms of changing women’s drinking behaviour in pregnancy. Training of practitioners appears to be an important means to improve their skills and enhance their confidence to screen and deliver ABIs, yet there have been reports that even after training some General Practitioners did not adequately deliver ABIs [43]. Despite midwives perceived confidence, our study found some evidence that they sometimes misunderstood the purpose and nature of ABIs.

During pregnancy, women are screened for different behavioural risk factors that may impact on healthy pregnancy outcomes. The findings of this study showed that some midwives felt that alcohol consumption was not particularly problematic for the majority of their clients. This is in contrast with current survey data which showed that about 25% of pregnant women in Scotland consumed alcohol whiles pregnant [44]. It is recognised that such self-reporting survey data may underestimate drinking during pregnancy because of poor recollection and the social stigma issues associated with the behaviour [12,13,45]. Although it is difficult to estimate the true prevalence of some of the conditions associated with prenatal alcohol exposure such as fetal alcohol syndrome in most populations, the ramifications for children damaged before birth by alcohol make the screening and ABI program an important one.

Midwives attitudes to alcohol use in pregnancy could play an important role in the extent of their involvement in alcohol intervention activities. This study found that midwives personal use of alcohol influenced their attitudes to drinking in pregnancy and to some extent determined the kind of advice they reportedly gave to pregnant women. Whilst midwives who did not drink alcohol completely condemned the behaviour of drinking in pregnancy, there were conflicting opinions from those who did drink alcohol themselves. Among those who did drink alcohol, some felt it was necessary for pregnant women to abstain while others felt there was nothing wrong with pregnant women drinking alcohol occasionally, albeit at sensible drinking levels. A study exploring nurses attitudes towards ABIs in primary care in the North-east of England, found that nurses who were hesitant about the alcohol intervention activities were those who used alcohol themselves [36]. It appears that health professionals’ personal drinking behaviour subtly influences their alcohol intervention activities. However, considering the underlying differences between service user groups that present to antenatal care and primary care services, the primary healthcare nurses’ attitudes to alcohol consumption might be more understandable. For example, among primary care populations it has been shown that drinking at low levels reduces the risk of coronary artery disease and ischemic stroke in men over 40 years old and women during the menopause [46]. However, in the antenatal care setting, a large body of evidence highlights that drinking during pregnancy poses risks for mother and the unborn child. It seems that positive changes in attitudes among midwives towards prenatal alcohol consumption might further enhance alcohol intervention activities in this setting.

### Implications for midwifery policy, practice and future research

Public health interventions are generally complex and context-dependent and their transferability between countries, localities or settings requires careful consideration. What has worked for one group may not necessarily work for another because people’s values, behaviours and priorities differ [47,48]. Although, screening and ABIs have been evaluated extensively in primary care, their utility is limited in antenatal care and emerging evidence of their usefulness in this setting is often from countries outside the UK. Ongoing evaluation would be required to improve the program in the Scottish antenatal setting in terms of raising the priority of ABIs in the first antenatal appointment. Also, this study has showed that when examining outcomes, for instance, uptake of ABIs by midwives, it is important to consider issues in the process of implementing the intervention.

Pregnancy is a period where women are generally motivated to change risky behaviours. Therefore, pregnant women who still continue to drink at risky levels possibly require more extensive interventions, for example extended brief interventions to build their confidence to change their drinking behaviour [4,10].

Alcohol brief interventions do not only mean giving advice about drinking. They have structure and may require a ‘motivational style’ drawn from motivational interviewing. The practice of motivational interviewing requires a high level of skills and extensive training [49]. Effective practical assessment tools that can measure the integrity of practitioners’ motivational interviewing skill, for example, the motivational interview treatment integrity code could be a useful tool to assess and improve midwives’ motivational interviewing practice [50]. Good motivational interviewing or person-centred communication skills might also help to overcome barriers such as lack of midwife-pregnant woman rapport and facilitate screening and ABIs delivery at the first antenatal appointment.

Knowledge of risks (for example, fetal alcohol syndrome) is known to induce drinking behaviour change in pregnancy [51]. When training midwives to screen and deliver ABIs emphasis should be placed on the severity of some of these adverse fetal conditions. This may enhance the urgency of alcohol intervention activities in antenatal care. Currently, there is consistent evidence of effects of heavy drinking on the fetus. However, the evidence relating to low-moderate drinking is still inconclusive and our study has highlighted that there may be still scepticism among some midwives. There is therefore a need for more robust studies to establish the threshold and specific fetal risks that could results from these levels of drinking and to enhance communication of research findings to health practitioners.

### Limitations

This study was conducted in only one Health Board in Scotland and represented the views of a relatively small number of midwives. The findings of the study although not generalisable to all midwives delivering ABIs, may still be of interest to those involved in implementing similar interventions in antenatal care settings in any country. Considering the approach for recruiting participants, it was possible that midwives who felt knowledgeable about screening and ABIs were more likely to participate in the study. Therefore, midwives who did not participate may have had different views.

Both individual interviews and focus groups were conducted. Individual interviews offered participants the privacy of sharing opinions that could not be easily discussed in a group setting, especially as the program was a policy initiative. However, the group setting facilitated the development of ideas and debate and moved the discussion into dimensions which individual interviews were unable to achieve. We were keen to carry out more focus groups but unfortunately, by the nature of their work, community midwives are geographically dispersed and it was logistically challenging to conduct more focus groups. Concerning the analysis, because only one focus group was carried out, we felt it was inappropriate to analyse the data separately from the individual interview data. As a result, the two sets of data were analysed together, retaining the individual quotes from the focus group.

## Conclusion

The findings highlight what midwives perceived to be important in alcohol intervention program in antenatal care. Midwives recognised the important and strategic role they could play in alcohol intervention activities in antenatal care. They felt they had been adequately supported to screen and deliver ABIs, yet they were uncertain whether pregnant women were optimally benefiting from the screening and ABI program. Particularly, as they indicated that very limited numbers of pregnant women have received the ABIs. Undoubtedly, pre-pregnancy preventative measures might offer more benefits in terms of reducing alcohol-related fetal harm. However, with a ‘captive’ audience in antenatal, routine screening and ABIs present an opportunity for midwives to create awareness about the effects of drinking in pregnancy and possibly elicit positive drinking behaviour change that may well have ripple effects beyond the current pregnancy.

## Competing interest

The authors declare that they have no competing interests.

## Authors’ contribution

All authors made substantial contribution to design, acquisition and analysis and interpretation of data. LD collected and transcribed the data, and contributed to the analysis of data. He also drafted the manuscript. HC and RJ were involved in the data analysis and revising the manuscript. All authors read and approved the final manuscript.

## Pre-publication history

The pre-publication history for this paper can be accessed here:

http://www.biomedcentral.com/1471-2393/14/170/prepub
